# Application of 4D printing in dentistry: A narrative review

**DOI:** 10.34172/japid.2024.003

**Published:** 2024-03-16

**Authors:** Yeshwanth Perambudhuru, Lata Goyal, Meghna Dewan, Ajay Mahajan, Prabhat Kumar Chaudhari

**Affiliations:** ^1^Periodontics Division, Department of Dentistry, All India Institute of Medical Sciences, Bathinda, Punjab, India; ^2^All India Institute of Medical Sciences, New Delhi, India; ^3^HP Government Dental College, Shimla, Himachal Pradesh, India; ^4^Division of Orthodontics and Dentofacial Deformities, Centre for Dental Education and Research, All India Institute of Medical Sciences, New Delhi, India

**Keywords:** 3D printing, Computers, Dentistry, Dynamic, Printing, Smart material, Technology, Three-dimensional, time

## Abstract

4D printing is an innovative digital manufacturing technology that originated by adding a fourth dimension, i.e., time, to pre-existing 3D technology or additive manufacturing (AM). AM is a fast-growing technology used in many fields, which develops accurate 3D objects based on models designed by computers. Dentistry is one such field in which 3D technology is used for manufacturing objects in periodontics (scaffolds, local drug-delivering agents, augmentation of ridges), implants, prosthodontics (partial and complete dentures, obturators), oral surgery for reconstructing jaw, and orthodontics. Dynamism is a vital property needed for the survival of materials used in the oral cavity since the oral cavity is constantly subjected to various insults. 4D printing technology has overcome the disadvantages of 3D printing technology, i.e., it cannot create dynamic objects. Therefore, constant knowledge of 4D technology is required. 3D printing technology has shortcomings, which are discussed in this review. This review summaries various printing technologies, materials used, stimuli, and potential applications of 4D technology in dentistry.

## Introduction

 4D printing is a cutting-edge technology that is very much needed in dentistry to adapt to dimensional changes in the human body. It originated by incorporating the fourth dimension, i.e., time, to pre-existing 3D technology or additive manufacturing. 3D printing is a state-of-the-art technology that relies on computer-aided digital models to produce personalized 3D objects. It has been used for nearly three decades in various industries, including dentistry. Applications in dentistry include plaster models, crowns, bridges, and dentures in prosthodontics, patient- and jaw-specific implants, alveolar preservation frameworks, alveolar ridge augmentation, maxillary sinus augmentation in periodontics, 3D appliances in orthodontics, 3D obturators, and 3D restorative and endodontic filling materials. With the advent of different techniques and materials, 3D printing is slowly replacing traditional techniques.^1^

 Although 3D printing technology offers many advantages, such as producing the most intricate structures of the human body, which are impossible to produce with conventional manufacturing models, these structures are static. They cannot change with the dynamic environment.^2^ In wound healing, the tissue is remodeled in the region at every stage, with extensive changes in the epithelium and connective tissue cells. This dynamic process is essential for modulating important functions of the body. Adequate bone height and width are required for implant placement to ensure good stability and retention and prevent adjacent vital structures’ perforation. 3D-printed scaffold material has been used for bone augmentation in deficient ridge regions. However, with dimensional changes in the alveolar ridge over time, we need a scaffold material and an implant body that can modify its shape over time. Thereupon, the property of responding to dynamic events can be incorporated into printed material using 4D printing.^3^

 Tibbitis^2^ first proposed 4D printing to produce dynamic materials that can change shape over time. 4D printing is a process in which a 3D-printed object is modified into a different structure under the influence of external stimuli. The 4D technology is only 3D with an additional time factor as the fourth dimension, i.e., movement over time. This is made attainable by clever manipulation of the properties of several materials, which can be altered gradually by external stimuli such as temperature, fluid, light, magnetism, field of energy, pH, ion concentration, glucose concentration, and enzymes. In dentistry, 4D printing technology plays a positive role in varied specialties where there is a need for advanced materials that adapt to changing environmental conditions. Polymerization shrinkage, microleakage, dimensional changes of a denture, constant changes in the shape of the dental ridge, higher risk of damage to vital structures during the placement of implants, and frequent repeat visits are some of the disadvantages in dentistry that can be offset by 4D printing.

 This overview highlights different printing techniques, materials, stimuli used, and applications of 4D printing technology.

## Literature search

 A literature search on EMBASE, MEDLINE via Ovid, Web of Science, Cochrane Library, Database of Abstracts and Reviews of Effects (DARE), and various clinical trial registries was conducted to include relevant literature published up to February 2023. All the articles retrieved during the search that studied 3D and 4D printing were evaluated. The literature search was restricted to English-language publications. The following combination of terms was searched: “4D printing” OR “3D printing”, (Four-dimensional printing), (Three-dimensional printing), (Dentistry) AND (Oral cavity), (4D printing technology) AND (3D printing technology), (Additive manufacturing) AND (Biomimetics), (Printing) (Printings) AND (3D dimensional), (Bioprinting) AND (Dentists). In addition, articles were manually searched.

## Comparing 4D printing with 3D printing

 4D-printed materials are superior to 3D-printed ones because of their ability to change in response to stimulus. Materials that change their morphology and function depending on the environment are essential for addressing many problems in the biomedical field.^4^ Both 3D and 4D materials are produced using additive manufacturing technology. 3D-printed materials are static and do not adapt to the environment over time, while the 4D manufacturing process meets the above requirements (Table 1).^5^

**Table 1:** Differences between 3D and 4D printing technologies

	**3D printing**	**4D printing**
Technique	Additive manufacturing technology with static materials	Additive manufacturing technology with smart materials
Dimensions	Three dimensions	Four dimensions (time is added)
Reaction to time and stimulus	Materials do not respond to time and stimulus	Smart materials transform after being subjected to external stimulus
Built process	Here, one layer is printed, and the next second layer is printed above the first layer	It is the same as 3D printing but with the additional advantage of using smart materials
Materials	Resins, ceramics, metals, polymers.	Smart, multi-materials
Flexibility	Stiff, firm, static materials are formed	Dynamic, flexible materials
Applications	Surgical guides, aligners, individual impression trays, splints, models, wax-up framework, crowns and bridges, implants, etc	Restorative materials, individual-specific implants, dentures, splints, local drug delivery systems, root canal filling materials, ridge-specific dentures, etc

## Various technologies commonly used for printing 3D/4D objects in dentistry

 Both 3D and 4D technologies use the same additive manufacturing (AM) to produce materials.^6,7^ AM is a manufacturing technique where the first product design is made in the computer, which controls the printing of that predetermined material layer by layer until the end product is formed. The formed layer’s design, time, and speed are digitally controlled here. Different technologies use different methods of manufacturing material layer by layer.

 These techniques include stereolithography (SLA), computed axial lithography, digital light projection, powder binders, photopolymer jetting, selective laser sintering (SLS), and fused deposition modeling (FDM).^8^

 These AM systems are operated to form a material layer in the x-y plane and then build up layer by layer in the z-plane direction.

###  1. Stereolithography 

 Developed by 3D Systems in 1986, SLA is an additive manufacturing process that allows elements to be produced using a computer-aided design file (CAD).^9^ The product’s internal and external design can be created by computer software using mathematical equations or scanned images or data sets from magnetic resonance imaging (MRI) or computed tomography. The ability to use data from scanned images makes manufacturing useful, particularly for various applications in the biomedical field, as they enable the production of patient-specific models, prototypes, or implants.^9,10^

 The production of 3D objects by SLA: Stereolithography has a computer-controlled laser source, an X-Y scanning mirror that controls the direction of the laser based on the XY geometry of the design, a tank containing liquid resin and a computer-controlled build platform that moves while an object is being printed.

 Initially, the building platform will be submerged in the tank of a depth equal to the first formed layer. After the product is fully printed, the excess resin is removed (usually with isopropyl alcohol). The product is then cured with ultraviolet light to improve its mechanical properties.^10^ One of the limitations of this technique is the low availability of resins. The ideal resin should solidify quickly when illuminated with light. In the resins used so far, polymerization occurs upon illumination, and the cross-links form glass-like networks. Many resins were developed later. Most available resins are based on three main properties: low molecular weight, multifunctional monomers, and highly cross-linked networks. Disadvantages of this technology are over-curing of resin, lack of surface smoothness, lower mechanical strength, and their irritating effect.^11^

 Dental products manufactured by SLA have poor mechanical properties. The integration of nanoparticles into the chemical bonding matrix improves the mechanical properties. Incorporating ceramic fillers (coupling agents) improves the stress distribution of the printed material, preventing it from breaking. In addition, antimicrobial agents are added to solve the problem of bacterial colonization of intraoral appliances. To summarize, SLA can be a fast, beneficial, and versatile approach for 3D dental printing.^7^

###  2. Fused deposition modelling

 It is the second best 3D printing technology after SLA and is comparatively less expensive. The production of 3D objects by FDM: The components of this printer are a continuous filament, which is the raw material. A filament spool is located on the side or back of the printer and is loaded into the extruder. The extruder’s job is to push the filament to the hot end, the heater heats the solid filament at the nozzle to achieve a semi-fluid state, and the nozzle is the final part of the print head, the build platform onto which the material is deposited.^7,12^

 An important detail in this method is the thermoplasticity of the filament, which allows the filaments to fuse during printing and cure at room temperature after printing. Accuracy also depends on the thickness of the filament, its diameter, and the speed at which it moves. Commonly, filaments used in FDM are composites such as polyvinyl chloride, polycarbonates, poly-sulfones, and low-melting-point alloys such as bronze filaments. The reason for the anisotropy of the material is the lower adhesion between layers, which is an advantage of this technique. FDM is used to print temporary dentures in dentistry due to its weak mechanical properties.^7^

###  3. Photopolymer jetting

 This technique uses two or more print heads, one for building the material and the other for the substrate. Printers with multiple print heads can print objects from multiple materials in various colors.^13^

 The production of 3D objects by photopolymer jetting: Photopolymerisable polymer is injected into the print head, which is later hardened to print the object. The injection print head moves in an X-Y plane across the platform to build up material. UV curing lamp immediately cures and hardens the injected material. The supporting structure will support the printed material. The build platform moves down to make space for the material to build up.^7,13^

 This technique can print many types of resins and waxes used in casting. The print head and the working platform can move in both directions and independently of each other. The high production speed, smooth surface of the product, and low cost are different advantages of this technique. Difficulty removing the backing material, skin irritation, the inability to sterilize the skin, and high cost are some disadvantages of this technique.^4^

###  4. Digital light projection

 Another photo-curing technology identical to the SLA process is digital light projection (Table 2). In digital light projection, materials are 3D-printed layer by layer using photo-curing liquid photosensitive resins. For the substrate, the first layer is first built on a platform. This building platform can be of two types depending on the position of the UV source: ascending or descending. While the material is being printed, the current image is visible on the transparent underside of the resin. To obtain this image, digital light projection uses a digital projection screen to view the image.^14^

**Table 2:** Differences between digital light projection and stereolithography

	**Digital light projection**	**Stereolithography**
Light source	Uses UV light	The UV laser beam is used
The intensity of the light source	Adjustable	Non-adjustable
Method of curing	Resin is cured from all sides at the same time until the completion of the product	The laser beam is moved from one point to the next until the final product.
Accuracy of the final product	Less accurate, so it is used for printing bigger parts with fewer details	More accurate
Speed	Since the product is fabricated from all sides, DLP is faster.	Slow

DLP, Digital light projection

 A digital micro-mirror display, equipped with thousands of tiny mirrors that project the entire layer at once in precisely the required dimensions, is used to reflect the light(Table 2).^7^

###  5. Powder binder printer

 Powder compound printers can print objects in full color and are compatible with various materials. The operating mechanism of a powder binding printer: It has a material hopper with a supply of powdered material that is used to print objects, a build platform on which the material is built, a re-coater that reapplies the powder layer each time a layer is formed, and an inkjet print head that drips liquid adhesive onto the powder to create a strong bond between the powder particles.^15^

 After the final product is molded, high-pressure air is used to remove excess powder. Depending on the material, finishing operations are carried out to increase strength, e.g., parts made of metal binders must be heat treated at a low melting temperature, and sandblasted parts are infiltrated with acrylic resin. There is no risk of encapsulation as the binder is sprayed at room temperature.^7,15^

 The materials commonly used are metals, ceramics, and metal alloys such as titanium, stainless steel, and copper. This technique is mostly used in dentistry to produce study impressions, as the product printed with this technology is fragile and inaccurate. Therefore, this technique is suitable for applications that do not require sterilization. With this technique, there is a risk of deforming the product because the parts printed without support can deform due to their weight caused by heating. This technology’s manufacturing process is messy because of the powder use. However, it has its advantages, such as being faster and cheaper, no support structures are required, and metal parts are produced with low roughness.^7^

###  6. computed axial lithography

 This revolutionary volumetric 3D printing technique uses the principle of computed tomography to create objects from photocurable resin in a single step. Basically, the thin overhanging structures built using 3D printing must be supported by a supporting structure. To overcome this problem, computer-assisted axial lithography does not require supporting structures to create complex designs as it is printed using multi-beam technology where light is directed in different directions to the material for polymerization. In contrast, polymerization is done layer by layer in other printing methods like Digital light projection (DLP) and SLA. This technique does not print layer by layer, which speeds up processing. This layer-less manufacturing process is influenced by computed tomography. In this technique, irradiating a rotating vat with resin photopolymer from multiple light patterns causes the polymer to solidify. This technique forms the structure in a shorter time.

 This 3D printing technique is limited to light-sensitive materials, which limits its ability to produce different materials. The scattered light, the viscosity of the resin, and the optical overlay all affect the accuracy of the product. As the light has to pass through the wall of the container, there is a certain amount of refraction, reflection, and attenuation. Therefore, these factors must be controlled to print accurate objects.^7^

###  7. Selective laser sintering 

 A powerful laser is used for sintering small powders of polymers into a solid structure. The components of an SLS 3D printer are a powerful laser to fuse the powder, a scanning system that directs the laser beam to the predetermined location depending on the design, a build chamber that stores and delivers the powder, and a roller that deposits the powder onto the previously formed layer. The sintering process occurs inside the build chamber.^16^

 Since the material is supported by the powder surrounding it, no support material is required for this SLS process. Nylon is the most common material used for SLS because it is strong, stiff, and inexpensive. Unlike other techniques, such as powder bonding, the product of SLP can be sterilized. This technique is also not expensive. Since the surrounding powder serves as a support, SLP does not require any support structures. SLP’s processing time is also shorter as there are no post-processing steps. However, there are health risks due to the powder used, and the initial set-up is costly.^7,16^

## Materials used for 3D/4D printing

###  1. Polymers 

 Vinyl polymers: Because of their flexibility, they are often used in dentistry. Vinyl polymers are made from vinyl monomers. These polymers are not suitable for medical and dental use, although they are biocompatible because they are biodegradable. This does not bode well for long-term use, mainly when used as implants, which usually require long-term stability. In 3D digital printing, vinyl polymers are used after their mechanical properties have been modified.^17^ An example of a vinyl polymer that is mostly chosen for the production of denture base materials is poly(methyl methacrylate), as it is easy to process, weightless, non-reactive in the oral environment, and has a high aesthetic quality. Despite these advantages, it has disadvantages, such as weak mechanical and poor surface properties. By adding some additives such as silica and alumina, the antibacterial property can also be integrated by adding TiO_2_.^7,17^

 Styrene polymers: Polystyrene and acrylonitrile-butadiene-styrene are two commonly used styrene polymers used in dental 3D/4D printing. Various properties of these materials are outlined in Table 3.

**Table 3:** Various properties of polystyrene and ABS (thermoplastic polymer)

**Polystyrene**	**ABS (thermoplastic polymer)**
It is transparent Amorphous structure with a smooth surface Good mechanical properties Easy to fabricate	High tolerance to heat Impact strength is better Rigidity to chemicals like styrene, acrylonitrile, and butadiene

ABS, Acrylonitrile butadiene styrene

###  2. Polyesters 

 PLA, PC, and PCL are forerunners among the countless available biocompatible polyesters due to their superior biocompatibility, mechanical properties, and easy printability. Upon hydrolytic degradation of the material, no toxic products are produced.^7,18^ Table 4 compares three polyesters, namely polycarbonates, polycaprolactone, and polylactic acid, regarding their applications, advantages, and disadvantages.

**Table 4:** Comparison of three polyesters, namely polycarbonates, polycaprolactone, and polylactic acid

**Polyesters**	**Polycarbonates**	**Polycaprolactone**	**Polylactic acid**
Applications	Ortho brackets Dentures Temporary crowns	Alveolar bone augmentation. Scaffolds for bone tissue engineering	Producing guides for surgical insertion of dental implants Temporary restorations
Advantages	Impact strength is appreciable Dimensionally stable Better electrical properties Lightweight Heat resistance Toughness	Good compatibility with biological tissue Slow degradation rate Unlike polyesters, there will be a minimum breakdown in acid Can be blended with a variety of polymers and hydrogels to improve its properties High flexibility Low melting point	Highly biocompatible material Flexible Physico mechanical property The better processability
Limitations	Easily attacked by hydrocarbons and bases Release of harmful substances like bisphenol A Yellowing tendency	no potential to initiate bone regeneration	Low heat resistance, so it can be used for high-temperature applications Rapid deformity is seen under high temperatures. Low tensile strength

###  3. Metal-based materials 

 All 3D/4D technologies that use metals use metal powder either as a raw material or bound inside a filament. Metals have benefits like resistance to wear and better mechanical properties. Metal-based products are used in medical applications more often because of their good mechanical and biocompatible properties. The latter property allows metal-based products to work efficiently without causing local or systemic side effects.^7^ Comparison of various properties of commonly used metals in 3D printing technology are summarized in Table 5.

**Table 5:** Various properties of commonly used metals in 3D printing technology

**Metals**	**Properties **
Vanadium steel	The first implant was made using vanadium steel. It has the disadvantage of the failure of implant function Mechanical and corrosion failure.
Stainless steel	To overcome the above limitations, stainless steel was introduced Stainless steel has more strength and better corrosion resistance Corrosion resistance is because of its chromium and nickel content The problem with a considerable amount of chromium and nickel is they can cause allergic reactions in some patients, which will further force a second surgery to remove those materials Manufacturing permanent implants is not advisable with stainless steel because of the chances of cracking, susceptibility to stress, and formation of deep pits on the surface
Titanium	Titanium replaced stainless steel due to its resistance to corrosion, biocompatible nature, high strength, and less weight Though Ti-6Al-4V has many advantages without surface treatment and coating, it cannot be used because of its poor wear resistance The toxicity of titanium alloy is because of the presence of aluminum and vanadium in it
Cobalt-based alloys	Cobalt-based alloys are used as a substitution for titanium alloy because of the high cost High wear and corrosion resistance Good mechanical properties Good tensile and yield strength. The presence of Cr, Mo, and Co favors the formation of a thin protective passive oxide film after exposure to body fluids Mostly, Cr oxides will be present

###  4. Ceramics 

 In daily dental practice, ceramics use has increased rapidly due to some of the properties of ceramics, such as high aesthetics, tactility, biocompatibility, electrical insulation, hardness, and high or low thermal conductivity, depending on the formulation. Ceramics are used in many fields, such as aerospace, automotive, energy, medical, chemical, and pharmaceutical industries. In the medical and dental sectors, ceramics are used for implants, surgical instruments, and diagnostic devices. For 3D printing of models, ceramics are used in binder jetting, powder sintering, nanoparticle jetting, and DLP. In all manufacturing processes, the ceramics must be fired to cure, and the systems require additional sintering steps before firing.^7^

###  5. Zirconia 

 Zirconia is an oxide ceramic. Once sintered, it is used for various applications in dentistry. It has good mechanical properties even at high temperatures. Materials such as aluminum oxide can be added to zirconia to improve its mechanical properties, and yttrium oxide can improve its electrical conductivity. Its biocompatibility and resistance to high temperatures make it the perfect material for dentistry. Zirconia can also be used to make implants, allowing good osseointegration with hard tissue. 3D printing technologies using zirconia include SLS, SLA, and inkjet printing, but the most common process available on the market is digital light processing. Zirconia also reduces inflammatory responses and plaque adhesion, inhibits microbial growth, and regulates fibroblast adhesion and proliferation.^19^

###  6. Alumina 

 Alumina is the cheapest oxide ceramic, available in various forms. The α-form is the most durable form. Various applications in dentistry include abutments, posts, wires, implants, and dentures. Aluminum oxide replaces metal alloys due to its high purity of almost ∼99.99%. Aluminum oxide is abrasion-resistant and compatible with the properties of biological tissue, but its bending strength is lower than zirconia. Zirconia can be added to aluminum oxide to enhance its mechanical properties.^20^ 3D printing systems that use alumina include material extrusion, material jetting, powder-binder jetting, plate lamination, SLA, and powder bed fusion, but the most commonly used system is SLA. The mechanical properties of alumina can be improved by increasing the grain size, decreasing the porosity, controlling the number of sintering cycles, time, and temperature, and adding stabilizing agents such as zirconium, magnesium, and chromium oxides.^7,20^

 4D printing is achieved by multi-material printing. In addition, smart materials that change over time can also be produced by 4D printing, which falls under single-material printing. To fully understand smart materials, one should know how external stimuli such as light, temperature, electricity, magnetism, pH, and chemical substances can cause changes in smart materials.

####  (i) Thermo-responsiveness

 4D materials can also be thermoactivated, meaning they can change shape with temperature changes. The smart materials used here undergo various thermal changes during the manufacturing process to become thermoreactive. This property is advantageous when these smart materials are used in restorative dentistry, where intraoral restorative materials undergo various thermal changes to adapt and prevent microleakage. Even in implant dentistry, implants can be inserted into small bone defects in response to a thermal stimulus. Thermoreactive materials can also be used in local drug delivery, with the controlled release of drugs based on the difference in body temperature. For example, some smart materials can store drugs when circulating in the body’s circulation but release the drug when it reaches the tumor site where the temperature is high. Shape-changing materials should be used instead of shape-memory materials to achieve this property. Shape-memory materials deform in response to a stimulus and return to their original shape once the stimulus is removed.^21^

 Thermo-responsive materials like isopropyl acrylamide, vinyl caprolactam, gelatin, collagen, soybean oil, Pluronic, and ether urethane are the most used materials in 4D printing.^21,22^

####  (ii) Moisture-responsiveness

 Materials that respond to moisture are of great interest because water/moisture is everywhere and stimulates these materials. The human body comprises nearly 75‒80% water, distributed both intracellularly and extracellularly. Therefore, water/moisture can be used as a stimulus to achieve transformations. Hydrogels are examples of moisture-sensitive materials that can inflate twice their actual volume due to their hydrophilicity. In addition, hydrogels exhibit high compressive strength. Hydrogels are typically placed in a hydrophilic environment, allowing them to take in moisture until saturation. The expansion of hydrogels can also be regulated by controlling the surrounding aqueous temperature. Various applications of moisture-sensitive materials include controlled delivery of drugs (through the property of differential swelling) and encapsulation of cells (when cells are transplanted into foreign body tissues, reactions occur due to immunity; to protect these transplanted cells, they are covered with a membrane called cell encapsulation). The disadvantages are that these smart materials do not react immediately, the mechanical properties are weak, and the product’s durability must be considered, as it has to swell and shrink several times.^22,23^

####  (iii) Photo-responsiveness

 Light can be used as a stimulus to change materials from one form to another. The basic mechanism of photosensitive materials is that they use light heat for transformation. The main advantage of using light as a stimulus is that, unlike a moisture stimulus, the reaction is rapid, and the materials change quickly and for a long time. To work effectively, photosensitive materials must be exposed to light. Light triggers transformation in different ways, such as changing size, shape, and charge generation. In pharmaceuticals, it is used for drug delivery, where irradiation at a specific wavelength can cause the capsule to burst open, resulting in targeted drug delivery. Photosensitive materials consist of azobenzene, stilbene, fulgide or diarylethene as well as photosensitive metal nanostructures.^24^

####  (iv) Magneto-responsiveness

 Magneto-active/responsive materials are substances that respond to magnetic fields. These materials are generally produced using nanomagnetic materials such as iron, cobalt, manganese, and nickel. In the current generation, magnetics has a wide range of applications in the biomedical field, ranging from MRIs to drug delivery. Breger et al^24^ used a remote control to apply a magnetic field to a microgripper after incorporating magnetic nanoparticles into hydrogels.

 Biologically stimulated materials (e.g., glucose) are another category to consider. For example, some materials respond to the body’s high blood glucose levels and can release insulin to control blood glucose levels. Self-awareness, self-reaction, shape memory, auto-repair, self-adjustment, and versatility are characteristics of smart materials.

## 4D printing in dentistry

 The introduction of 4D printing in 2012 created curiosity in the manufacturing sector, especially in the medical and dental fields. Biomedicine involves diagnosis, drug delivery, tissue engineering, and implants. 4D printing offers 3D-printed structures the ability to respond dynamically to different stimuli by changing their shape, size, form, and structure over time. 4D printing technology is enabling greater innovation, particularly in restorative dentistry. The 3D dental scanner has made the dentist’s job hassle-free and convenient.^25^

 Research studies on 4D printing in dentistry will continue to increase to create prosthetic materials, aligners, templates, and orthodontic brackets that can be modified according to the patient’s specific response to the stimulus. This will provide superior treatment and cleverly resolve the obstacles in dentistry today. Tooth loss due to the extraction of poorly predicted teeth or orthodontic treatment can lead to bone loss, making it difficult to place implants due to insufficient dimensions. With the help of 4D printing technology, dental implants with good biological and mechanical properties can be produced like natural teeth. The patient’s artificial teeth are now adjusted with constant changes in the jaw ridge shapes according to the patient’s different habits. 4D-printed dental implants have properties such as dimensional changes, which could help avoid marginal leakage of dental implants if the implants have the good property of dimensional changes.^26^

 Most dental implants are made of titanium alloys. Despite their advantages, these implants experience problems such as hypersensitivity and surface wear. Substituting titanium with smart polymers not only increases the strength of the implant but also improves the tunable properties, offers good elasticity, is cost-effective, takes less time to manufacture, has low density, is easy to process, is more flexible to manufacture, and is environmentally friendly. Therefore, applications ranging from tissue support to permanent implants can be easily manufactured with higher viability and reliability.^7^

 Scaffolds can be fabricated using 4D printing and used in periodontal defects in soft and hard tissues for periodontal regeneration. This scaffold promotes the formation of cementum, bone, and PDL and aids in reattachment in the periodontal defect. 4D-printed scaffolds can also be used for alveolar maintenance to prevent bone loss.^27^

 Iatrogenic errors are observed in implant surgery where there is a risk of damaging adjacent vital structures, such as the inferior alveolar nerve and the maxillary antrum, which can be observed in conventional implant placement. 4D-printed membranes can be placed beneath the dental implant to protect vital structures like the maxillary sinus or inferior alveolar nerve. If the implant is processed with a smart material that is modified in the apical part and has a soft base that adapts to the surrounding tissue, damage to vital structures can be significantly reduced or avoided. Moreover, no additional complex surgeries, such as sinus augmentation, are required to make room for the implant when smart materials are used instead of conventional implants. In regenerative dentistry, stem cells can be used to regenerate lost or defective hard and soft tissue using 4D-printed scaffolds as supporting structures.^28^ 4D-printed objects in the form of dental implants or a scaffold can be fused with stem cells to grow into a natural tooth. Its use can be extended in TMJ and maxillofacial surgeries using 4D-printed materials to replace cartilage to compensate for articulation and occlusion.^27^

 Endodontics is an area where most failures are due to microleakage, which 4D-printed smart materials can control. Another drawback in endodontic treatment is broken files, which are currently difficult to restore. However, with the introduction of smart materials that change shape with various stimuli, they can be easily restored.^7^ Smart materials can also be used in orthodontics as implants that improve the efficiency of ligatures and wires that help move teeth into a specific position.^26^

 Confocal laser scanning microscopy is a potent laser technology with significant growth in recent years. It uses parameters such as time and stained substances to study the functional activities of a living cell. Compared to conventional 3D microscopic technology to scan live tissues, carious lesions, and bone defects, confocal microscopy has better contrast and no blurring with samples of different thicknesses. It provides good resolution and examination of fluorescent-labeled samples without sectioning. Eventually, this 3D design can be upgraded to a 4D structure by adding time or position.^29^

 Various methods exist to replace lost teeth, such as dentures, bridges, crowns, and implants. Three-dimensional scanning technologies and CAD and manufacturing are now used in the design of dentures and processing. Both denture bases and denture teeth can be machined and bonded together with various bonding agents.^7^

 These dentures have higher reproducibility and produce dentures with greater accuracy. They can overcome the inconvenience faced by the patient in traditional denture fabrication and reduce problems after insertion. No matter how good the denture made with 3D technology is, the alveolar ridge is reabsorbed over time, affecting the denture’s fit. With 4D technology, the denture can adapt to the current ridge shape.

 Smart materials can be used as restorative materials when sealing root canals to prevent microleakage and reinfection, prevent microbial film formation, and allow for easy restoration when needed. Ni-Ti archwires are commonly used in orthodontics, with many advantages and disadvantages. Replacing these Ni-Ti wires with smart materials will reduce many disadvantages, such as frequent reactivation. The superior aesthetics, improved modulus of elasticity, improved physicochemical properties, and auto-adjusting properties of the wire make this material more appropriate for patients and practitioners.^7^

 Normally, the restorative material bonding with the tooth structure should be chemical to avoid microleakage and achieve a firm adhesion. However, the tooth structure has to go through many steps, such as etching/adhesive bonding, where the prepared site is contaminated with saliva. However, with the availability of smart restorative materials, firm bonding with mechanical rather than chemical retention is possible.^28^

 Drug delivery systems are gaining much attention in the current situation as they increase the concentration of the drug in the desired area without having a systemic effect. The advantages of these drug delivery systems include adequate drug concentration at the desired site, increased bioavailability, longer residence time at the site, fewer side effects, no harmful chemical reactions with the drugs, and reduced frequency of drug intake.^28^

 The principle of a local drug delivery system is to concentrate the drug taken at a specific site in the body and minimize the systemic complications of drugs. The main advantages of this system are that an acceptable drug concentration can be achieved near the desired site, there are no systemic effects, there is no risk of overdose, and the drug does not spread to adjacent vital organs. Various smart materials that slowly release drugs when activated by thermo-, moisture-, photo-, and pH-dependent reactions are produced using 4D printing technology.^22^

## Summary

 In recent decades, 3D printing technology has developed rapidly and offers comprehensive support in various areas, especially in the dental field. 3D-manufactured implants, crowns, bridges, various restorative materials, and orthodontic appliances are of great value as they are custom-made for individual patients.^30,31^ Applications in periodontics include regeneration of periodontal tissues, sinus augmentation, ridge augmentation, implants, and surgical guides for implant placement.^32^

 Despite the many advantages, materials made with 3D printing technology are static and cannot adapt to changing external and internal stimuli and patient growth. Some commonly observed problems in dentistry with 3D products are microleakage, loss of stability of dentures due to progressive resorption of the alveolar ridge over time, and frequent patient recalls for device activation and retreatments.^7^

 4D printing technology has progressed over the past decades and has positively affected various fields. In 4D printing, time is integrated into traditional 3D printing technology, through which a transformation of the 3D material occurs with time when it is stimulated with external stimuli. 4D technology adds time as a fourth dimension to 3D technology. Both 3D and 4D printing technologies use the same AM technique to construct products. Materials are made layer by layer until the final product has a predetermined design controlled by a computer. Materials used for 3D printing include many resins, polymers, metals, and ceramics. 4D printing, on the other hand, uses smart materials that can respond to different stimuli.

 Different techniques are used for processing, and this technology has different methods for printing models layer by layer. Among these technologies, the most common techniques for biomedical applications are bucket polymerization, material jetting, powder binding, and powder bed fusion. The use of 4D will benefit various areas of medicine and dentistry, most notably tissue reconstruction, targeted drug delivery, implants, new diagnostic devices, and artificial substitutes that mimic human tissue, such as stents, prostheses, and scaffolds.^27^ 3D printing technology has simplified the dentist’s work. First, 3D scanners are used for scanning, and this scanned patient data is passed to the 3D printing technology, which uses various smart materials and smart printing technologies to produce the final 4D product. The difficulties encountered in traditional work can be overcome by 4D technology.^2^

## Conclusion

 Despite making complex things easier, 3D printing technology has its disadvantages. It can be overcome by 4D printing technology by manufacturing things and adapting them to the local environment in the medical and dental fields. However, 4D technology is limited to a few specialties in dentistry. Further research work and clinical trials should be done on applying 4D printing technology in different aspects of dentistry to expand knowledge on 4D printing.

## Competing Interests

 The authors declare no competing interests.

## Consent for Publication

 Not applicable.

## Data Availability Statement

 Not applicable.

## Ethical Approval

 Not applicable.

## Funding

 None.
